# The association of GPR85 with PSD-95-neuroligin complex and autism spectrum disorder: a molecular analysis

**DOI:** 10.1186/s13229-015-0012-5

**Published:** 2015-03-13

**Authors:** Eriko Fujita-Jimbo, Yuko Tanabe, Zhiling Yu, Karin Kojima, Masato Mori, Hong Li, Sadahiko Iwamoto, Takanori Yamagata, Mariko Y Momoi, Takashi Momoi

**Affiliations:** Department of Pediatrics, Jichi Medical University, 3311-1 Yakushiji, Shimotsuke-shi, Tochigi 3290498 Japan; Medical Research Center, International University of Welfare and Health, 2600-1 Kitakanemaru, Ohtawara, 3248501 Japan; Department of Pediatrics, Shengjing Hospital of China Medical University, 36 Sanhao Street, Heping District, Shenyang, 100004 Liaoning China; Medical Biochemical Genetics, Harvard Medical School, 25 Shattuck Street, Boston, MA 02115 USA; Division of Human Genetics, Center for Molecular Medicine, Jichi Medical University, 3311-1 Yakushiji, Shimotsuke-shi, Tochigi 3290498 Japan

**Keywords:** Autism spectrum disorder, ASD, GPCR, ER stress, Synaptic receptors, GPR85

## Abstract

**Background:**

Autism spectrum disorder (ASD) has a complex genetic etiology. Some symptoms and mutated genes, including *neuroligin* (*NLGN*), *neurexin* (*NRXN*), and SH3 and multiple ankyrin repeat domains protein (*SHANK*), are shared by schizophrenia and ASD. Little is known about the molecular pathogenesis of ASD. One of the possible molecular pathogenesis is an imbalance of excitatory and inhibitory receptors linked with the NLGN-PSD-95-SHANK complex via postsynaptic density protein/Drosophila disc large tumor suppressor/zonula occludens-1 protein (PDZ) binding. In the present study, we focused on *GPR85* as a candidate gene for ASD because the C-terminal amino acid sequence of GPR85 [Thr-Cys-Val-Ile (YCVI)] is classified as a type II PDZ-binding motif, and *GPR85* is a risk factor for schizophrenia. GPR85 is an orphan receptor that regulates neural and synaptic plasticity and modulates diverse behaviors, including learning and memory. While searching for molecules that associate with GPR85, we found that GPR85 was associated with postsynaptic density protein (PSD)-95 linked with NLGN in the brain.

**Methods:**

We examined the proteins that associate with the C-terminal sequence of GPR85 by pull-down assay and immunoblot analysis and searched for a mutation of the *GPR85* gene in patients with ASD. We used immunostaining to examine the intracellular localization of mutated GPR85 and its influence on the morphology of cells and neurons.

**Results:**

The C-terminal sequence of GPR85 interacted with PSD-95 at PDZ1, while NLGN interacted with PSD-95 at PDZ3. Two male patients with ASD from independent Japanese families possessed inherited missense mutations at conserved sites in *GPR85*: one had T1033C (M152T) and the other had G1239T (V221L). These mutations were located in a domain related to G protein interaction and signal transduction. In contrast to wild-type GPR85, mutated GPR85 was more preferentially accumulated, causing endoplasmic reticulum stress, and disturbed the dendrite formation of hippocampal neurons.

**Conclusions:**

GPR85 associated with the PSD-95 linked with NLGN, which is related to ASD. GPR85 carrying the mutations detected in ASD patients disturbed dendrite formation that could be the candidate for molecular pathogenesis of ASD through the associated NLGN-PSD-95 receptor complex.

**Electronic supplementary material:**

The online version of this article (doi:10.1186/s13229-015-0012-5) contains supplementary material, which is available to authorized users.

## Background

Autism spectrum disorder (ASD) has a complex genetic etiology. Of the genes that have been shown to confer susceptibility to ASD, many are involved in synaptic adhesion and formation, including *neuroligins* (*NLGN*) *3* and *4* [1], SH3 and multiple ankyrin repeat domains protein (*SHANK*) *3* [2], contactin-associated protein-like (*CNTNAP*) *2* [3], and cell adhesion molecule (*CADM*) *1* [4]. For instance, NLGNs are postsynaptic cell adhesion proteins that interact with neurexins (NRXN) on the presynaptic membrane, and they are required for synapse maturation [5]. NRXN-NLGN interactions induce differentiation of γ-aminobutyric acid (GABA) and glutamate postsynaptic specializations [5]. The extracellular domain of NLGN displays *trans*-cell adhesion activity [6], and its intracellular domain of NLGN associates with postsynaptic density protein (PSD)-95 via individual postsynaptic density protein/Drosophila disc large tumor suppressor/zonula occludens-1 protein (PDZ) domains [7]. One of possible causes of pathogenesis is an imbalance of receptors including excitatory and inhibitory receptors linked with the NLGN-PSD-95-SHANK complex via PDZ binding [5].

In addition to synaptic adhesion molecules, alterations in G-protein-coupled receptor (GPCR; GPR) signaling are likely to be associated with ASD pathogenesis. GPRs include the GABA and serotonin [5-hydroxytryptamine (5-HT)] receptors - which are involved in numerous neuronal processes, including the regulation of synaptic formation and transduction [8]. We recently found that mutations in the *GPR37* gene, located in the ASD linkage locus 9 (*AUTS9*) on chromosome 7q31, are associated with the manifestation of ASD [9]. Some mutations apparently had a causative, deleterious effect, and other mutations appeared to confer susceptibility to ASD under predisposing conditions [9].

Mutations in *NRXN1*, *NRXN2*, *NLGN2*, *NLGN4*, and *SHANK3* genes related to ASD have been also found in schizophrenia patients [10-13]. There are some overlapping symptoms between schizophrenia and autism, particularly the negative symptoms (poverty of speech and volition, social withdrawal, and blunt affect) [14,15]. Pharmacological treatments have also suggested that GPRs are the common target molecules in psychiatric disorders including ASD and schizophrenia [14]. Thus, the pathogenesis of ASD and schizophrenia may have some common molecular basis [15], but little is known about them. We focused on *GPR85* located in the *AUTS9* locus in the 7q31 region [16] that harbors *GPR37* [17].

*GPR85* is involved in predisposing patients to a high risk of schizophrenia; two *GPR85* SNPs in moderate linkage disequilibrium have been associated with schizophrenia [18], but mutations in *GPR85* have not been found in patients with schizophrenia. GPR85 is an orphan receptor that is involved in determining brain size, regulating neural and synaptic plasticity, and modulating diverse behaviors (including learning and memory) [18]. The amino acid sequence of GPR85 is identical in all vertebrates [19,20]. GPR85 has type II PDZ-binding motif, Thr-Cys-Val-Ile (YCVI), at its C-terminal region [21]. During the study on the common molecular basis in the psychiatric disorders including schizophrenia and ASD, we found that the C-terminal sequence of GPR85 was linked with NLGN and PDZ proteins including PSD-95 in the brain.

In the present study, we examined the interaction between GPR85 and PDZ proteins linked with NLGN and mutations of *GPR85* in ASD patients. Here, we show two independent missense mutations in the *GPR85* gene of Japanese patients with ASD and describe the mutated GPR85-induced endoplasmic reticulum (ER) stress and deleterious effect on the dendrite formation of neurons.

## Methods

### Participants

Lymphocytes were obtained from 72 unrelated Japanese ASD patients with autism or a pervasive developmental disorder not otherwise specified. Their conditions were diagnosed according to criteria outlined in the Diagnostic and Statistical Manual of Mental Disorders, Fourth Edition (DSM-IV). Written informed consent was obtained from the parents of all patients. The patients included 57 males and 15 females, aged 3 to 23 years, with intellectual levels that varied from normal to severely disabled. Four probands had siblings with ASD, and others were sporadic. Control (*N* = 622) samples, including male (*N* = 381) and female (*N* = 241) samples, were obtained from healthy Japanese adult volunteers after they provided written, informed consent. We also obtained the DNA of 200 Caucasian patients from the Autism Genetic Resource Exchange Consortium (Cure Autism Now, Los Angeles, CA). AGRE samples included 172 males and 28 females; all were familial cases. Caucasian control DNA samples were obtained from the Coriell Institute (Camden, NJ).

Written informed consent was obtained from the parents of all subjects, subjects themselves in capable cases, and control subjects. The study was approved by the Bioethics Committee for Human Gene Analysis of Jichi Medical University (approval number, 11–52, 12–87).

### Direct sequencing analysis

Genomic DNA was extracted from peripheral blood lymphocytes or lymphoblasts using a standard method described by the manufacturer. We used polymerase chain reaction (PCR) primers to amplify each exon and its boundaries in the *GPR85* regions. The primers are listed in Additional file 1: Table S1. PCR products were purified by passing them through micro-concentrating centrifugal filter columns (Millipore, Bedford, MA, USA). Sequencing reactions were performed using the Applied Biosystems Dye-Terminator Kit and analyzed on an ABI Prism 3730 DNA Sequencer (Applied Biosystems, Foster City, CA, USA).

### Site-directed mutagenesis and DNA construction

The full length of GPR85 cDNA was isolated from human cDNA library (Stratagene, La Jolla, CA, USA). To generate GPR85 mutants, site-directed mutagenesis were performed by using QuikChange II Site-Directed Mutagenesis kit (Stratagene) with the following primers: for mutation of M152T, sense primer: 5′-CTCTGTCTGTGGCCA**C**GGCATTTCCCCCGGTTTT-3′; antisense primer: 5′-AAAACCGGGGGAAATGCC**G**TGGCCACAGACAGAG-3′; for mutation of V221L, sense primer: 5′-GAAGCCAGTCCAGTTT**T**TAGCAGCAGTCAGCCA-3′; antisense primer: 5′-TGGCTGACTGCTGCTA**A**AAACTGGACTGGCTTC-3′. Underlined nucleotides were mutated sites. Mutations were confirmed by DNA sequencing.

The enzyme-digested PCR DNA fragments corresponding to full-length GPR85 and full-length GPR85-deltaC for the pcDNA4/TO/myc-His expression vector (Invitrogen, Carlsbad, CA, USA) or pcDEF3-FLAG vector [4], GPR85-C and GPR85-deltaC were subcloned into pGEM-T easy vector (Promega, Madison, WI, USA) using GPR85 PCR primers as follows: forward primer: 5′-GGTACCATGGCGAACTATAGCCATGC-3′ for full-length GPR85 and full-length GPR85-deltaC, and reverse primer: 5′-GGTACCGTATAACACAGTAAGGTTCC-3′ for full-length GPR85-myc-His; or reverse primer: 5′-GGTACCGAGGTTCCTTGGTAAC-3′ for full-length GPR85-deltaC-myc-His, reverse primer: 5-GAATTCTCATATAACACAGTAAGGTTCC-3 for FLAG-full-length GPR85; or reverse primer: 5-GAATTCTCAAGGTTCCTTGGTAAC-3 for FLAG-full-length GPR85-deltaC, forward primer: 5′-GGATCCTCCAGGTTACCAAGGGAACC-3′ for GPR85-C and GPR85-deltaC, and reverse primer: 5′-GGATCCTCATATAACACAGTAAGGTTC-3′ for GPR85-C; or reverse primer: 5′-GGATCCTCAAGGTTCCTTGGTAAC-3′ for GPR85-deltaC. Full-length GPR85 and GPR85-deltaC were subcloned into *Kpn*1 sites of the pcDNA4/TO/myc-His expression vector, and GPR85-C and GPR85-deltaC fragments were subcloned into *Bam*H1 sites pGEX4T-3 vector (Pharmacia, Buckinghamshire, UK). FLAG-GPR85 wild type and mutants in pGEM-T easy vector were subcloned into *Eco*R1 sites pcDEF3-FLAG vector.

The experiments were approved by the Genetic Modification Safety Committee of Jichi University (approval number, 11–52) and were carried out under the jurisdiction of the Ministry of Education, Culture, Sports, Science and Technology.

### Animal care

We followed the Fundamental Guidelines for Proper Conduct of Animal Experiments and Related Activities in Academic Research Institutions under the jurisdiction of the Ministry of Education, Culture, Sports, Science and Technology. All protocols for animal handling and treatment were reviewed and approved by the Animal Care and Use Committee of Jichi University (approval numbers, 11-157, 12076) and by the International University of Health and Welfare (approval numbers, D1008, 10118).

### Cell culture

Primary culture of hippocampal neurons was prepared from mouse wild-type embryos at embryonic day (E) 18 as previously described [22]. Neurones maintained in AraC (5 μM)-containing medium for 2 days (days *in vitro* (DIV) 3 to 5). Briefly, hippocampalis were dissected in Hepes-buffered saline solution and dissociated by adding 10% trypsin and 1% DNase and incubating for 15 min. Neurons were plated at a density of 2 × 10^4^ cells/cm^2^ on 0.05% polyethylenimine-treated, 1.2 mm-diameter cover slips (Matsunami, Osaka, Japan) and incubated in Neurobasal™ medium with B-27 supplement (Invitrogen) and 2% fetal bovine serum. Cultures were maintained at 37°C in 5% CO_2_. Half of the growth medium was exchanged with fresh medium once a week. Neurons with developed dendritic arbors were used for the experiments. COS and C2C5 [23] cells were cultured in α-minimum essential medium (MEM) with 10% fetal bovine serum (FBS) at 37 °C in a humidified atmosphere of 5% CO_2_.

### Transfection

Neurons (cultured for 7 days (DIV 7)) and C2C5 cells were transfected with pcDNA4 plasmid containing wild-type or mutated *GPR85* (M152T or V221L)-myc-his, pcDEF plasmid containing FLAG-wild-type, mutated *GPR85* (M152T or V221L), or wild-type, mutated *GPR85* (M152T or V221L)-deltaC and PSD-95-GFP [24] constructs. Transfections were performed with lipofectamine 2000, and then cells were incubated for 28 to 48 h.

### Immunoblot analysis

C2C5 cells were lysed in buffer (20 mM Tris-HCl, 150 mM NaCl, 1 mM EDTA, 10% glycerol, 1% Triton X-100) and protease inhibitor or buffer A in ProteoExtract® Native Membrane Protein Extraction Kit according to the manufacturer’s protocol (Merck Millipore, Darmstadt, Germany). Separated proteins were transferred to nitrocellulose transfer membrane by electroblotting. Nonspecific binding was blocked with 0.5% nonfat dry milk with 0.1% Triton X-100 in PBS. After centrifugation, 40 μg of total protein was subjected to SDS-PAGE (7.5% to 12%). Then, blots were incubated overnight at 4 °C with mouse anti-His (MBL, Nagoya, Japan); mouse anti-CHOP (Santa Cruz Biotech. Inc., Dallas, Texas, USA) levels were assessed by adding alkaline phosphatase-conjugated and goat anti-rabbit or anti-mouse immunoglobulins (Promega) and detecting the signals generated in reactions with nitro blue tetrazolium and 5-bromo-4-chloro-3-indolyl-1-phosphate. Data from three experiments were scanned and analyzed for quantification with Image J software (National Institutes of Health). Results compared with wild type were analyzed using Student’s *t*-test (*P* < 0.05 was considered statistically significant).

### Immunostaining

Cells were fixed in 4% paraformaldehyde (PFA) or methanol in PBS. After they were incubated with blocking reagent (PBS containing 1% goat serum, 1% skim milk, and 0.1% Triton X-100) for 1 h at room temperature, they were then subjected to the immunostaining using mouse anti-His, rabbit anti-His (MBL, Nagoya, Japan), mouse anti-FLAG, rabbit anti-FLAG (Sigma), rabbit anti-eIF2α (Cell Signaling Technology, Inc., Danvers, MA, USA), mouse anti-CHOP (Santa Cruz Biotechnology), mouse anti-MAP2 (Sigma), mouse anti-PSD-95 (Thermo Fisher Scientific, Rockford, IL, USA), and rabbit caspase-3 [25]. Alexa-Fluor-488- and Alexa-Fluor-568-conjugated secondary antibodies against mouse and rabbit IgG were purchased from Molecular Probes (Eugene, OR, USA). Nuclei were detected by Hoechst 33342 (Molecular Probes). Immunofluorescence was viewed using a confocal laser-scanning microscope (CSU-10, Yokogawa Electric Co., Tokyo, Japan), a Leica TCS SP5 confocal microscope, and Leica AF6000 Modular Systems (Leica Microsystems, Wetzlar, Germany).

The percentages of C2C5 cells that showed apoptotic morphology and CHOP, P-eIF2, and caspase-3 positivity were determined from a total of 200 cells that expressed wild type or 200 cells that expressed mutant GPR85 at 28 to 48 h after transfection. The percentage neurons with long dendrites were determined from a total of 100 neurons that expressed wild type or 100 neurons that expressed mutant GPR85 at 28 to 48 h after transfection. The experiments were repeated three times. The values represent averages of the percentages of the number of cells obtained in three experiments. Results were analyzed using Student’s *t*-tests (*P < 0.05 **P < 0.01 was considered statistically significant).

### GST pull-down assay

For *in vitro* pull-down assays, glutathione S-transferase (GST; estimated MW; 26 kDa) and the GST fusion proteins GST-GPR85-C including amino acids (SRLPREPYCVI) (estimated MW; 27.5 kDa) and GST-GPR85-deltaC (lacking the PDZ-binding sequence, YCVI) (estimated MW; 27.1 kDa) were purified by Glutathione Sepharose 4B (GE Healthcare Biosciences, Buckinghamshire, UK) from the extract of *Escherichia coli* containing each plasmid. GST fusion proteins bound to Sepharose beads were incubated with lysates from the brain or COS cells transfected with GFP-PSD-95 and GFP-SAP102 for 2 h at 4°C. The beads were washed three times with lysis buffer and then eluted with 50 mM Tris-HCl (pH 8.0) containing 20 mM reduced glutathione. The bound proteins were then separated by SDS-PAGE and detected by immunoblotting using the following primary antibodies: rabbit anti-SAP102 (Synaptic systems, Goettingen, Germany), rabbit anti-PSD-95 (Millipore), mouse anti-Mupp1 (Becton Dickinson, Franklin Lakes, NJ, USA), mouse anti-Neuroligin (Synaptic Systems), mouse anti-GFP (Roche Diagnostics Schweiz, Rotkreuz, Switzerland), and rabbit anti-GST (Santa Cruz Biotechnology).

## Results

**Figure 1:**
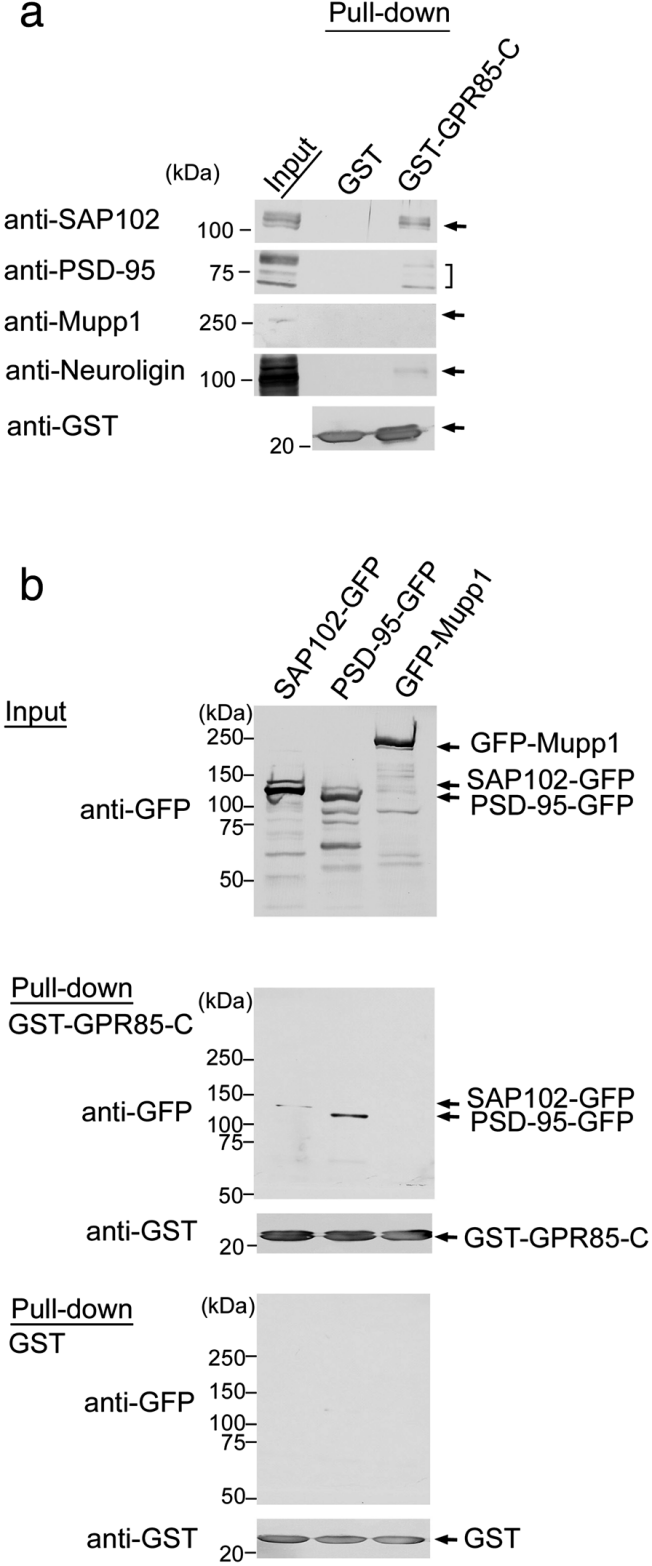
**Pull-down and immunoblot analysis for the proteins associating with GPR85-C. (a)** GPR85-C-associating proteins in the mouse brain. The extracts from the mouse brains were subjected to the pull-down assay using GST-GPR85-C and immunoblot analysis. GPR85-C band with PSD-95, SAP102, and NLGN, but not with Mupp1 in the extracts from mouse brain. **(b)** The interaction between GPR85-C and PSD-95 or SAP102. The extracts from COS cells transfected with GFP-PSD-95, GFP-SAP102, and GFP-Mupp-1 were subjected to the pull-down assay using GST-GPR85-C. GPR85-C interacted with GFP-PSD-95 and weakly interacted with GFP-SAP102, but not with Mupp1. GST, glutathione S-transferase; PSD, postsynaptic density protein.

The C-terminal amino acid sequence of human and mouse GPR85 (YCVI) is classified as a type II PDZ-binding motif [21]. We employed a pull-down assay and immunoblot analysis using specific antibodies to examine whether the C-terminal sequence of GPR85 (GPR85-C) interacts with PDZ proteins in the mouse brain. In mouse brain extracts, GPR85-C interacted with PSD-95, SAP102, and NLGN, but not with Mupp1 (Figure 1a). As NLGN binds PSD-95 and SAP102 through its C-terminal PDZ-binding domain, we examined the interaction between GPR85-C and GFP-PSD-95 or GFP-SAP102 expressed in COS cells using pull-down assay and immunoblotting with anti-GFP (Figure 1b). GPR85-C interacted more strongly with GFP-PSD-95 than with GFP-SAP102.

**Figure 2:**
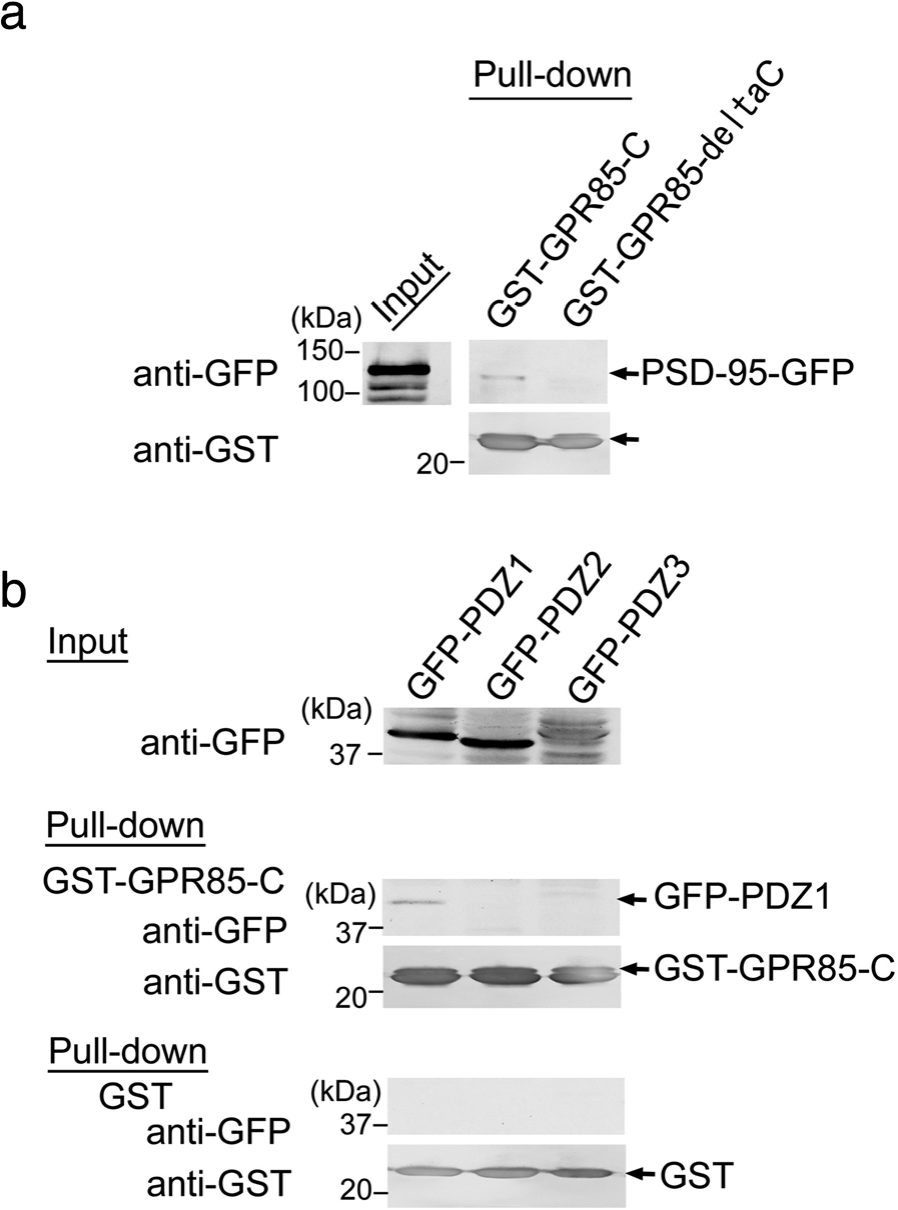
**Identification of PDZ domain of PSD-95 interacting with GPR85-C. (a)** PSD-95 was associated with GST-GPR85-C, but not with GST-GPR85-deltaC lacking YCVI. **(b)** GST-GPR85-C interacted with PDZ1 of PSD-95, but not with PDZ2 nor PDZ3. GST, glutathione S-transferase; PDZ, postsynaptic density protein/Drosophila disc large tumor suppressor/zonula occludens-1 protein; PSD, postsynaptic density protein.

Next, we examined the binding domain in detail during the interaction between GPR85 and PSD-95 (Figure 2). GPR85-C lacking YCVI (GPR85-deltaC) did not interact with PSD-95 (Figure 2a). PSD-95 has three PDZ domains, PDZ1, PDZ2, and PDZ3. GPR85-C interacted with PDZ1, but not with PDZ2 or PDZ3 (Figure 2b). Therefore, GPR85 likely associated with the NLGN-PSD-95 complex related to pathogenesis of ASD through individual PDZ domains.

**Figure 3:**
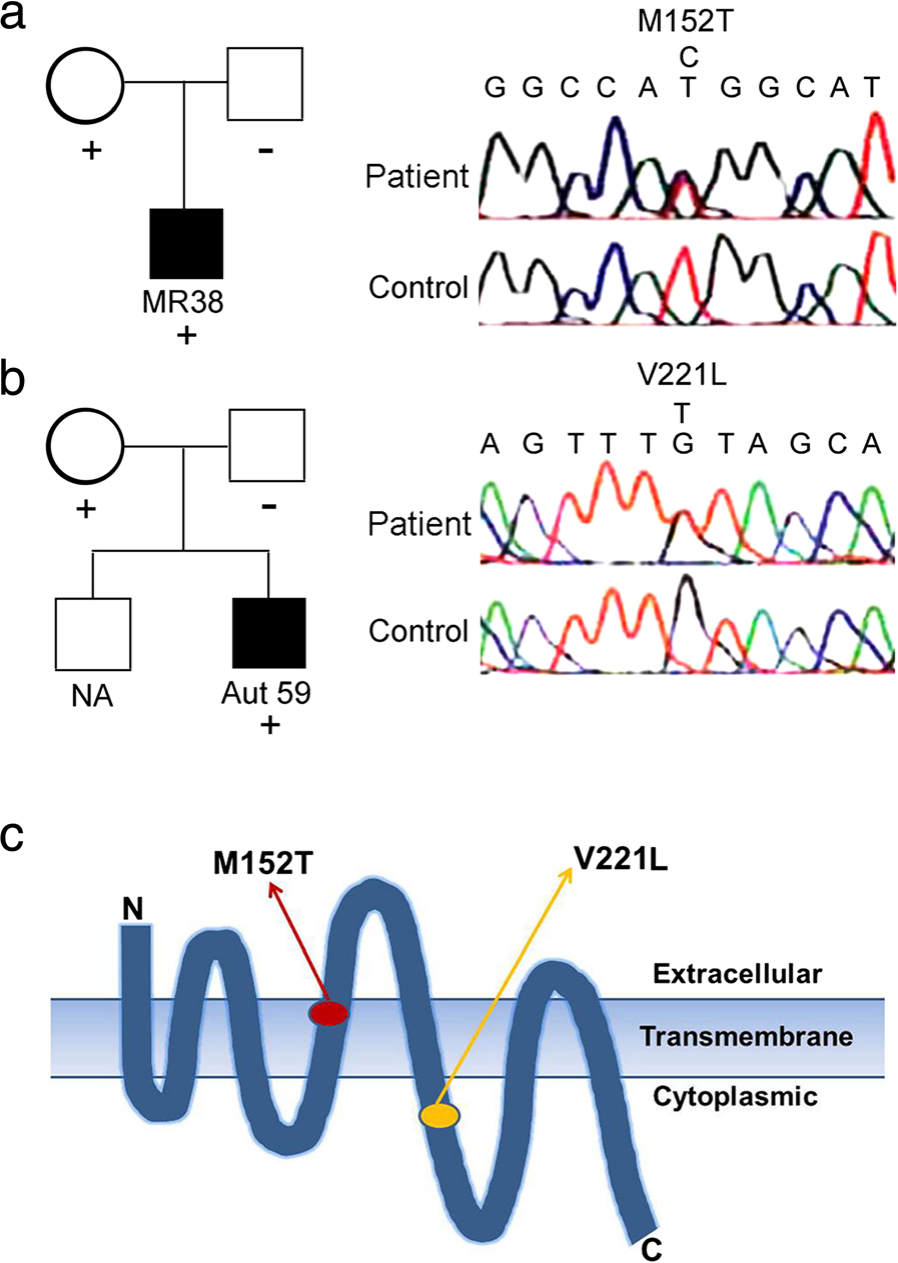
***GPR85*** **mutations detected and family trees of the patients with each base substitution. (a)** M152T in *GPR85* of patient MR38 and his family member. **(b)** V221L in *GPR85* of patient Aut59 and his family member. NA, not available. **(c)** The position of two mutations, M152T and V221L, in the human GPR85 molecule. Arrows indicate the position of M152T (red) and V221L (yellow).

To examine the common molecular basis of schizophrenia and ASD, we searched for GPR85 mutations in Caucasian and Japanese ASD patients. In two males among 72 Japanese patients, we identified two base alterations in *GPR85* that caused amino acid substitutions: one was T1033C (M152T) and the other G1239T (V221L) (Table 1; Figure 3a, b). Each of these mutations was also detected in the patients’ apparently unaffected mothers (Figure 3a, b). No sample was available to test the brother of the patient with V221L. Neither base alteration was found in 189 Caucasian ASD samples and 622 healthy Japanese volunteers. Individuals with either the M152T or V221L mutation in *GPR85* presented with similar clinical phenotypes; they exhibited ASD with mild intellectual disability, the ability to speak some simple words, and no epilepsy. We also detected two single base substitutions (Table 1), but they were different from the SNPs (rs56080411 and rs56039557) reported as risk factors of schizophrenia [18].

**Table 1:** **Results of the analysis of** ***GPR85***

**Base change**	**AA change**	**Patients**	**Control**	**Report**
***Japanese patients***	**(** ***n*** **= 72)**			
c.830C > T	p.G84G	(C/C) 71/72		SNP
		(C/T) 1/72		
		(T/T) 0/72		
c.938 C > T	p.Y120Y	(C/C) 71/72		SNP
		(T/C) 1/72		
		(T/T) 0/72		
c.1033 T > C	p.M152T	(T/C) 1/72	0/622	Fa-, Mo+
c.1239G > T	p.V221L	(G/T) 1/72	0/622	Fa-, Mo+, Bro NA
***Caucasian patients***	**(** ***n*** ** = 189)**			
c.830C > T	p.G84G	(C/C) 111/151		SNP
		(C/T) 37/151		
		(T/T) 3/151		
c.938C > T	p.Y120Y	(C/C) 139/187		SNP
		(T/C) 44/187		
		(T/T) 4/187

*GPR85* is transcribed into four RNA isoforms that, like other GPCRs, encode seven transmembrane (TM) domains. These four GPR85 RNA isoforms are registered in GenBank as NM_001146265.1, NM_018970.6, NM_001146266.1, and NM_001146267.1. The two mutations found in this study, M152T and V221L, were located in exon 3, which is present in all four RNA isoforms. One mutation was located in the fourth TM domain and the other in the cytoplasmic region adjacent to the fifth TM domain (Figure 3c).

**Figure 4:**
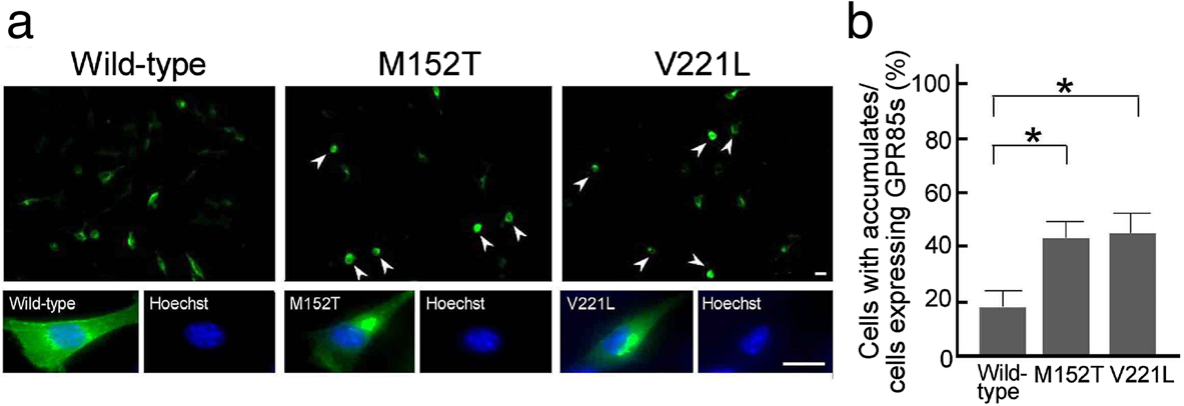
**The accumulation of the mutated GPR85 (M152T) and GPR85 (V221L) in C2C5 cells. (a)** Intracellular accumulates of wild-type and the two mutated GPR85. Green, full-length GPR85 (wild-type or mutants)-Myc-His; blue, Hoechst. **(b)** Percentages of cells showing accumulates were determined by counting cells expressing wild-type and mutated GPR85 at 28 h after transfection. Scale bars, 25 μm.

We focused on the intracellular localization of the wild-type and mutated GPR85 proteins expressed in C2C5 cells. Both the exogenous and endogenous GPR85 proteins were localized mainly in the ER [see Additional file 2: Figure S1]. Compared to wild-type GPR85, GPR85 mutants accumulated in the ER more frequently (Figure 4a). ER accumulation of GPR85 was observed in 43% of cells with the M152T mutation and 45% of cells with the V221L mutation, but in only 17% of cells with normal GPR85 (Figure 4b).

**Figure 5:**
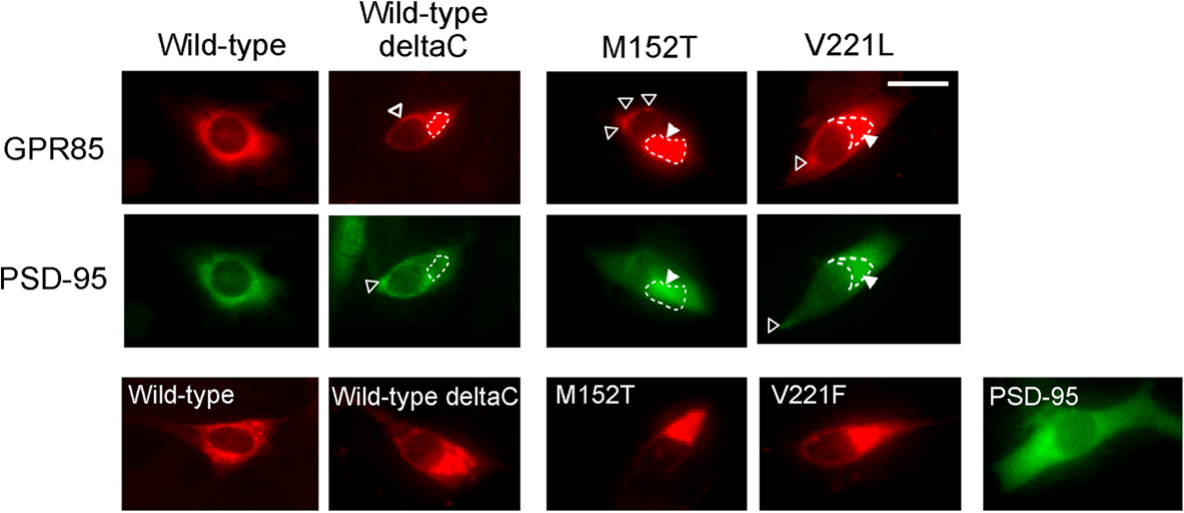
**Co-localization of the wild-type and mutated GPR85 and PSD-95.** Co-localization of wild-type GPR85, GPR85-deltaC, GPR85(M152T) or GPR85(V221L), and PSD-95 was examined in C2C5 cells at 28 h after transfection. Red, FLAG-Full-length GPR85 (wild-type, deltaC, or mutants); green, PSD-95-GFP; some of the GPR85-deltaC, GPR85(M152T), GPR85(V221L) did not co-localize with PSD-95. Area enclosed by broken lines, aggregates of GPR85; closed arrowheads, GPR85 of co-localized with PSD-95; open arrowheads, GPR85 or PSD-95 not colocalized with PSD-95 or GPR85, respectively. Scale bar, 20 μm. PSD, postsynaptic density protein.

We examined the co-localization of wild-type or mutated GPR85 (M152T, V221L) with PSD-95 in C2C5 cells. Wild-type GPR85, but not GPR85-deltaC, co-localized with PSD-95 (Figure 5). In contrast to wild-type GPR85, some of the mutated GPR85 did not co-localize with PSD-95 (Figure 5).

**Figure 6:**
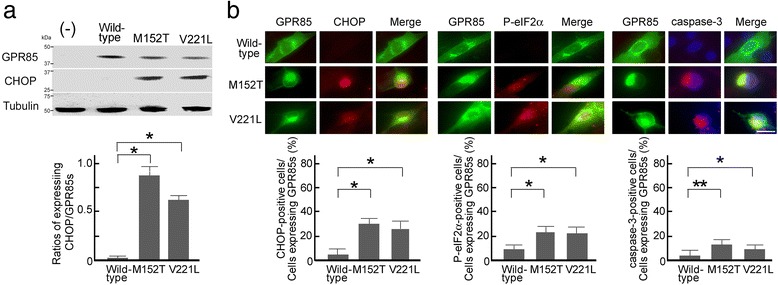
**The mutated GPR85-molecule-induced ER stress in C2C5 cells. (a)** Up-regulation of CHOP protein induced by the wild-type and the mutated GPR85. **(b)** Detection of activation of ER stress markers, CHOP and eIF2α phosphorylation, and activation of caspase-3 by immunostaining (upper panel) and percentages of their positive cells (lower panel). Green, GPR85; red, Chop and eIF2α phosphorylation (p-eIF2α) at 28 h after transfection and activated caspase-3 at 32 h after transfection; blue, Hoechst. Scale bar, 20 μm. The percentage of the cells positive for CHOP, p-eIF2α, and activated caspase-3 in the cells expressing wild-type and the two mutated GPR85. Error bars indicate standard error of the mean (*P < 0.05, **P < 0.01).

Cells that expressed the mutated GPR85 protein also exhibited ER stress markers, including CHOP up-regulation (Figure 6a, b), eIF2α phosphorylation, and activated caspase-3 (associated with apoptosis; Figure 6b). Thus, the mutated GPR85 localized to the ER and induced ER stress.

**Figure 7:**
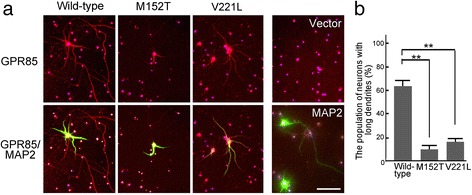
**Morphologies of dendrites of neurons expressing wild-type and mutated GPR85. (a)** Immunostaining of hippocampal neurons (DIV 9) expressing the wild-type and the mutated GPR85. Isolated hippocampal neurons from mice embryos (E18) were transfected with His-tagged wild-type and mutated GPR85 (M152T or V221L) at DIV 7 and cultured and immunostained with anti-His (red) and MAP2 (green) at DIV 9. Hoechst, blue. Scale bar, 50 μm. **(b)** Percentages of cells with long dendrites (1,000 μm). Error bars indicate standard error of the mean (**P < 0.01).

We examined the influence of GPR85 mutants on the morphology of murine hippocampal neurons. Cultured embryonic hippocampal neurons were transfected with wild-type or mutant *GPR85* and allowed to develop for 2 days *in vitro*. The neurons were then classified into three categories based on dendrite length by immunostaining with MAP2, a dendritic marker: long dendrites (>1,000 μm), short dendrites (<1,000 μm), and no dendrites (<100 μm) (Figure 7 and Table 2). We found that neurons expressing mutated GPR85 (M152T, V221L) had shorter dendrites than neurons expressing wild-type GPR85. Furthermore, neurons expressing GPR85 mutants exhibited the decreased numbers of the dendritic branching points [see Additional file 3: Figure S2].

**Table 2:** **Percentages of the neurons expressing three types of dendrites in the neurons expressing GPR85**

**Types** ^**a**^ **(9 DIV** ^**b**^ **)**	**Wild-type**	**M152T**	**V221L**
Neurons with long dendrites, >1,000 μm	63.5 ± 9.0%	9.2 ± 4.8%	15.9 ± 7.0%
Neurons with short dendrites, <1,000 μm	31.7 ± 4.9%	13.5 ± 5.1%	20.5 ± 4.8%
Neuron with no dendrites, <100 μm	4.8 ± 1.6%	77.3 ± 4.6%	63.6 ± 4.5%

^a^Total dendritic length/cell; ^b^DIV = days *in vitro*.

**Figure 8:**
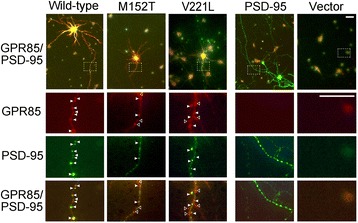
**Co-localization of wild-type GPR85 or GPR85 mutants and PSD-95 in neurons.** Co-localization of wild-type GPR85, GPR85(M152T) or GPR85(V221L), and PSD-95 was examined in hippocampal neurons (DIV 9) at 2 days after transfection. Red, wild-type GPR85 and GPR85 mutants (M152T and V221L)-Myc-His; green, PSD-95-GFP. Scale bars, 20 μm. Closed arrowheads, punctum of GPR85 co-localized with PSD-95 at synapse; open arrowheads, punctum of GPR85 not co-localized with PSD-95 at synapse. PSD, postsynaptic density protein.

We also examined the localization of wild-type and mutated GPR85 on the dendrites of neurons. The wild-type GPR85 punctate stainings co-localized with PSD-95, a postsynaptic marker, on the dendrites and in the soma, but less mutated GPR85 (M152T, V221L) punctate stainings were detected on the dendrites, and some of them did not co-localize with PSD-95 (Figure 8 and Additional file 4: Figure S3).

## Discussion

### Mutations in GPR85 and ASD

The primary amino acid sequence of Gpr85 is identical in human, monkey, rat, and mouse species [16,19]. Therefore, the M152 and V221 amino acids are conserved among all vertebrates. We found the M152T or the V221L substitution in the *GPR85* gene of 72 Japanese ASD patients and not in the 189 Caucasian ASD patients or in the 632 control Japanese samples (Table 1). The frequency of the mutation in *GPR85* in the Japanese patients with ASD seems to be quite similar to that of the mutation in other genes related to ASD. For instance, the frequency of *SHANK3* mutation is in up to 2% to 3% of individuals with ASD [26]. Moreover, T1033C (M152T) and G1239T (V221L) mutations were not associated with any polymorphisms or SNPs, suggesting that the M152T or V221L mutations were likely to be associated with ASD pathogenesis.

The M152T and V221L mutations were also detected in each patient’s asymptomatic mother. We previously observed this inheritance pattern, in which the ASD-associated variant allele appears in an asymptomatic mother and in her symptomatic male offspring, in our previous study on *MBD1* and other ASD-associated gene variants [27]. These findings, and the higher prevalence of ASD in males than in females, suggest that some unknown protective mechanism might be present in females that carry ASD-associated genes [27]. Other studies of pedigrees that harbor mutations in *SHANK3*, *CNTNAP2*, *MECP2*, *MBD1*, or *CADM1* have also reported that unaffected parents of either gender can carry an ASD-associated mutation [2-4,27,28]. The fact that ASD-related genotypes can be inherited from nonsymptomatic mothers or fathers supports the theory that ASD mutations are dominantly transmitted, with low penetrance in high-risk families [29].

### Impaired NLGN-PSD95-GPR85 receptor complex and dendrite formation

GPR-mediated signaling requires activation of the associated heterotrimeric G proteins. GPR85 is an orphan receptor; thus, little is known about its physiological function and signal transduction pathways. However, the M152T and V221L mutations are located in GPR85 domains that involve signal transduction and interaction with G proteins, suggesting that these mutations cause deleterious loss-of-function effects. Moreover, two software applications, the Mutation Taster and ‘SIFT’, suggested that the M152T amino acid change has a greater impact on protein function than the V221L amino acid change.

NLGN and CADM1/CNTNAP2 interact with PSD-95 and MUPP1 via their PDZ-binding sequences, respectively [24,30]. Mutations in these genes have been found in ASD patients [1,3,4], suggesting that the impaired function of these two distinct complexes is associated with the pathogenesis of ASD. GPR85-C was associated with both PSD-95 and NLGN in the brain (Figure 1). Similar to NLGN, GPR85-C interacted with PSD-95 through YCVI, a type II PDZ-binding motif, but not with MUPP1 (Figures 1 and 2). Furthermore, GPR85-C interacted with PSD-95 via PDZ1 (Figure 2), whereas NLGN binds PSD-95 via PDZ3. Therefore, GPR85 likely forms a complex with NLGN through a different PDZ domain of PSD-95.

In contrast to wild-type GPR85, some of the mutated GPR85 as well as GPR85-deltaC did not co-localize with PSD-95 in C2C5 cells and on the dendrites of neurons (Figures 5 and 8), suggesting that mutated GPR85 interacted more weakly with PSD-95 than wild-type GPR85, although the missense mutation is not located in the PDZ-binding sequence, which is necessary for interaction with PSD-95. Mutated GPR85-deltaC or GPR85-deltaC as well as the mutated GPR85 induced CHOP up-regulation [see Additional file 5: Figure S4], suggesting that, in addition to mutations themselves, weak interaction with PSD-95 causes accumulation of mutated GPR85 in the ER, resulting in the ER stress.

Compared to the neurons expressing wild-type GPR85, the neurons expressing mutated GPR85 exhibited shorter dendrites (Figure 7), the decreased number of GPR85 puncta, and an altered distribution of the mutated GPR85 on dendrites (Figure 8). These results suggest that the GPR85 mutations cause a trafficking defect due to a lack of interaction with PSD-95, resulting in impaired function of the NLGN-PSD-95-GPR85 complex on the dendrites.

However, impaired dendrite formation or synaptic formation has not been observed in the brain of *Gpr85*-deficient mice [18], suggesting that loss of function alone does not affect the physiological function of neurons. Mutated forms of GPR85 accumulated preferentially in the cells (Figures 5 and 6), suggesting that these mutations cause not only loss of function, but also the accumulation of mutated GPR85 and ER stress (Figure 6), leading to defects in their trafficking (Figure 8) and impaired dendrite formation (Figure 7). Blocked dendrite formation may cause the complex impairments in synaptic function that have been observed in patients with ASD who harbor *GPR85* mutations. This hypothesis will be studied in future *in vitro* culture experiments and using *Gpr85*(M152T) knock-in mice.

### Common molecular target for ASD and schizophrenia

Impaired synaptic functions are shared with other psychiatric disorders including schizophrenia. Some of the mutations of the genes related to ASD, including *NRXN1*, *NRXN2*, *NLGN2*, *NLGN4*, *SHANK3*, and *SynGAP*, have been also found in the schizophrenia patients [10-13]; for instance, two *de novo* mutations (R1117X and R536W) were identified in the *SHANK3* gene of two families of schizophrenia [13]. GPR85 is a risk factor for schizophrenia, suggesting that GPR85 could be related to the molecular pathogenesis of not only ASD but also schizophrenia via interaction with PSD-95 or SAP102.

Thus, the putative ligand of GPR85 and its signaling pathways may be a common molecular target for ASD and schizophrenia as described in [10-15]. We will investigate our hypothesis regarding the molecular pathogenesis of ASD and mutated GPR85 in future studies that employ *Gpr85* (M152T) knock-in mice.

## Conclusions

GPR85 interacted with PSD-95, associating with NLGN related to ASD. Two independent GPR85 mutations, T1033C (M152T) and G1239T (V221L), were found in male autism patients from Japanese families. Mutated GPR85 preferentially accumulated, causing ER stress and disturbing the dendrite formation of hippocampal neurons. Thus, mutated GPR85 could be associated with the molecular pathogenesis of ASD, and GPR85 may be a common molecular target for ASD and schizophrenia.

## Additional files

Additional file 1: Table S1. Primers sequences of GPR85. These primers (5 sets) were used for the nucleotide sequence analysis of GPR85. The fragment length and annealing temperature for each PCR product are indicated in this Table S1. Additional file 2: Figure S1. The localization of endogenous GPR85 in C2C5 cells. The GPR85 protein was detected by immunohistochemical staining methods using rabbit anti-GPR85. GPR85 was mainly localized in ER (rough ER). Scale bar indicates 20 μm. Additional file 3: Figure S2. Sholl analysis of hippocampal neurons transfected wild-type and mutated GPR85. In contrast to wild-type GPR85, the mutated GPR85 exhibit a decreased level of dendritic complexity in DIV 9. The *x*-axis indicates distance from the origin; the *y*-axis indicates the number of dendritic intersections. Error bars indicate SEM. Neurons transfected were wild-type GPR85 (*n* = 16), GPR85 (M152T) (*n* = 16), and GPR85 (V221L) (*n* = 17), and they were examined by Sholl analysis, which was performed using the NIH ImageJ (Sholl Analysis Plugin). Analysis parameters were as follows: starting radius, 1 μm; ending radius, 75 μm; radius step size, 2 μm; radius span, 1 μm; span type, median. Additional file 4: Figure S3. The localization of endogenous GPR85 in hippocampus neurons. The GPR85 and PSD-95 was detected by immunohistochemical staining methods using rabbit anti-GPR85 (red) and mouse anti-PSD-95 (green). Hoechst, blue. In the dendrites, GPR85 was mostly co-localized with PSD-95 at spines. Scale bar indicates 50 μm. Additional file 5: Figure S4. The effect of PDZ-binding domain on the localization in C2C5 cells and ER stress. Localization of FLAG-full-length GPR85-deltaC, GPR85(M152T)-deltaC, and GPR85(V221L)-deltaC protein were detected by immunohistochemical staining using mouse or rabbit anti-FLAG. They did not co-localize (arrows) with PSD-95-GFP and induced ER stress, detected by mouse anti-CHOP. Scale bars indicate 20 μm.
