# Supplementary material for: The association of GPR85 with PSD-95-neuroligin complex and autism spectrum disorder: a molecular analysis
Source: Mol Autism. 2015 Mar 13;6:17. doi: 10.1186/s13229-015-0012-5 (PMC4360946; doi:10.1186/s13229-015-0012-5)
Supplement: Additional file 1: Table S1. — Primers sequences of GPR85. These primers (5 sets) were used for the nucleotide sequence analysis of GPR85. The fragment length and annealing temperature for each PCR product are indicated in this Table S1. [file 13229_2015_12_MOESM1_ESM.pdf]

Table S1. Primers sequences of *GPR85*

Primer sequence	Products	Annealing temperature
1F TGAGGCTGTATCCTTATCCTC	419 bp	55°C
1R GTATAGAAGCGGTGATGGGC		
2F TCCACACTGCTTTCATGCTC	479 bp	56°C
2R GGTGTTTGCATTTTGCCTG		
3F TGGCTAGCAGGATTTGGAAG	444 bp	54°C
3R CAATTGCTCAGCAGAGAAGG		
4F TCTGTATGCTGATTGGCCTG	487 bp	55°C
4R CTGACCAATGATGGGTGATG		
5F CATTCAGACAATGCATCTTCAG	284 bp	54°C
5R GACTTTTAATTTCCCCACCGG		
