# Supplementary figures and images for: The association of GPR85 with PSD-95-neuroligin complex and autism spectrum disorder: a molecular analysis
Source: Mol Autism. 2015 Mar 13;6:17. doi: 10.1186/s13229-015-0012-5 (PMC4360946; doi:10.1186/s13229-015-0012-5)

**Figure S1**

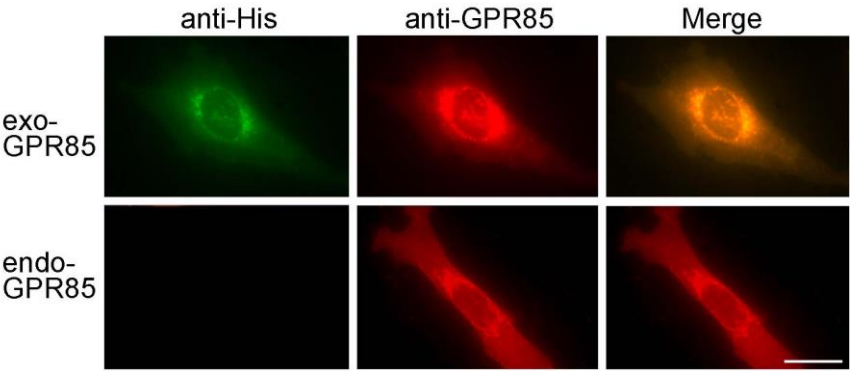

Supplement: Additional file 2: Figure S1. — The localization of endogenous GPR85 in C2C5 cells. The GPR85 protein was detected by immunohistochemical staining methods using rabbit anti-GPR85. GPR85 was mainly localized in ER (rough ER). Scale bar indicates 20 μm. [file 13229_2015_12_MOESM2_ESM.doc]

Figure S2

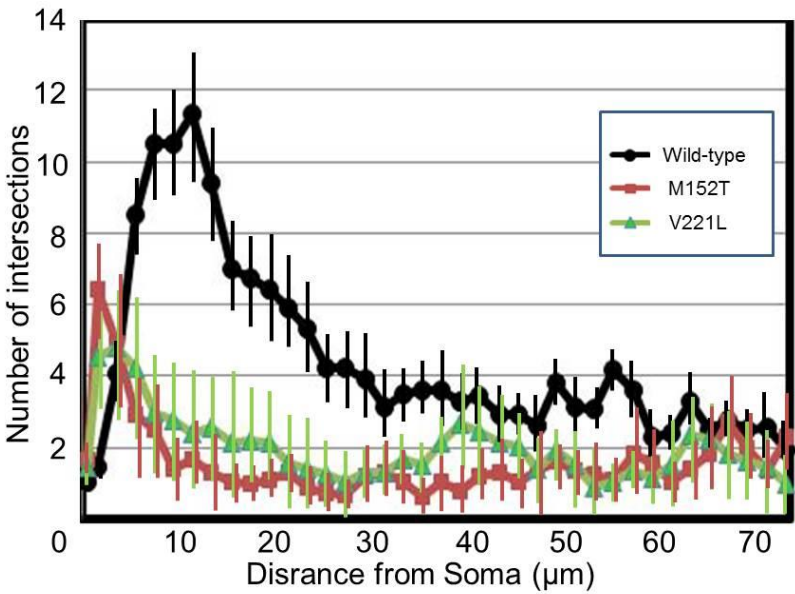

Supplement: Additional file 3: Figure S2. — Sholl analysis of hippocampal neurons transfected wild-type and mutated GPR85. In contrast to wild-type GPR85, the mutated GPR85 exhibit a decreased level of dendritic complexity in DIV 9. The x-axis indicates distance from the origin; the y-axis indicates the number of dendritic intersections. Error bars indicate SEM. Neurons transfected were wild-type GPR85 (n = 16), GPR85 (M152T) (n = 16), and GPR85 (V221L) (n = 17), and they were examined by Sholl analysis, which was performed using the NIH ImageJ (Sholl Analysis Plugin). Analysis parameters were as follows: starting radius, 1 μm; ending radius, 75 μm; radius step size, 2 μm; radius span, 1 μm; span type, median. [file 13229_2015_12_MOESM3_ESM.doc]

**Figure S3**

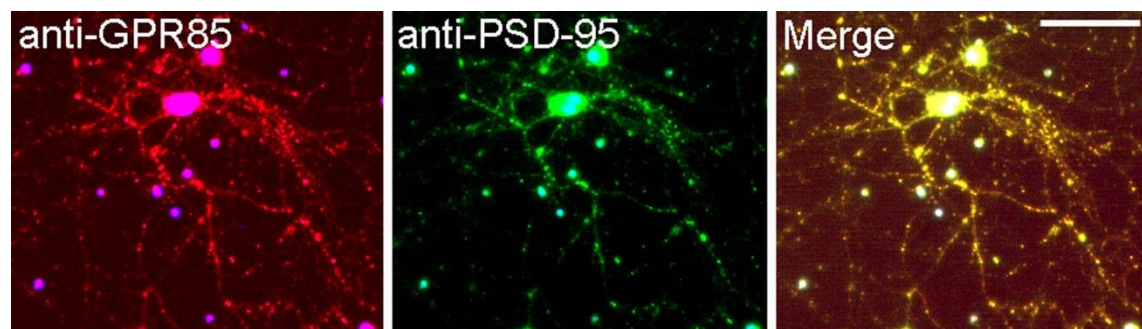

Supplement: Additional file 4: Figure S3. — The localization of endogenous GPR85 in hippocampus neurons. The GPR85 and PSD-95 was detected by immunohistochemical staining methods using rabbit anti-GPR85 (red) and mouse anti-PSD-95 (green). Hoechst, blue. In the dendrites, GPR85 was mostly co-localized with PSD-95 at spines. Scale bar indicates 50 μm. [file 13229_2015_12_MOESM4_ESM.doc]

Figure S4

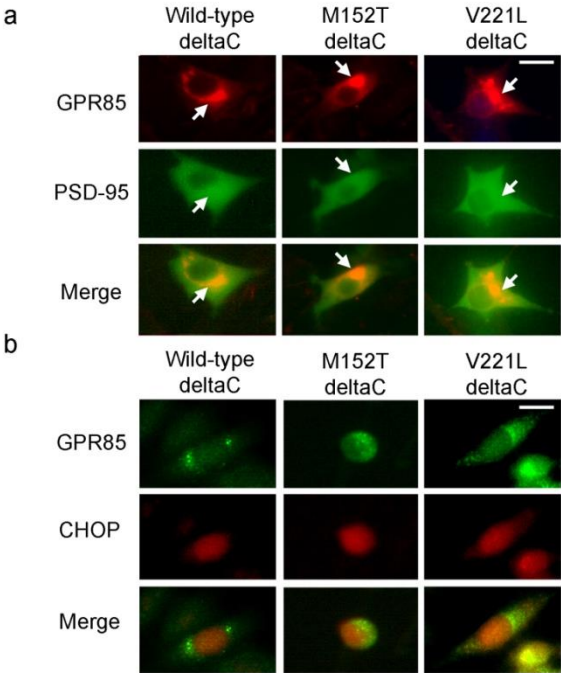

Supplement: Additional file 5: Figure S4. — The effect of PDZ-binding domain on the localization in C2C5 cells and ER stress. Localization of FLAG-full-length GPR85-deltaC, GPR85(M152T)-deltaC, and GPR85(V221L)-deltaC protein were detected by immunohistochemical staining using mouse or rabbit anti-FLAG. They did not co-localize (arrows) with PSD-95-GFP and induced ER stress, detected by mouse anti-CHOP. Scale bars indicate 20 μm. [file 13229_2015_12_MOESM5_ESM.doc]
